# S-Score: A Scoring System for the Identification and Prioritization of Predicted Cancer Genes

**DOI:** 10.1371/journal.pone.0094147

**Published:** 2014-04-07

**Authors:** Jorge E. S. de Souza, André F. Fonseca, Renan Valieris, Dirce M. Carraro, Jean Y. J. Wang, Richard D. Kolodner, Sandro J. de Souza

**Affiliations:** 1 Institute of Bioinformatics and Biotechnology, São Paulo, Brazil; 2 Center for Cell Therapy and Regional Blood Center, Department of Clinical Medicine, Faculty of Medicine, University of São Paulo, Ribeirão Preto, Brazil; 3 Brain Institute, UFRN, Natal, Brazil; 4 International Research Center, CIPE/AC Camargo Cancer Center, São Paulo, Brazil; 5 Moores-UCSD Cancer Center and Department of Medicine, University of California San Diego School of Medicine, San Diego, California, United States of America; 6 Ludwig Institute for Cancer Research, San Diego Branch, Department of Medicine and Cellular and Molecular Medicine, Moores-UCSD Cancer Center and Institute of Genomic Medicine, University of California San Diego School of Medicine, San Diego, California, United States of America; Tel Aviv University, Israel

## Abstract

A new method, which allows for the identification and prioritization of predicted cancer genes for future analysis, is presented. This method generates a gene-specific score called the “S-Score” by incorporating data from different types of analysis including mutation screening, methylation status, copy-number variation and expression profiling. The method was applied to the data from The Cancer Genome Atlas and allowed the identification of known and potentially new oncogenes and tumor suppressors associated with different clinical features including shortest term of survival in ovarian cancer patients and hormonal subtypes in breast cancer patients. Furthermore, for the first time a genome-wide search for genes that behave as oncogenes and tumor suppressors in different tumor types was performed. We envisage that the S-score can be used as a standard method for the identification and prioritization of cancer genes for follow-up studies.

## Introduction

The availability of different “omics” technologies and the recent development of next generation sequencing have brought new perspectives to the field of cancer research [1]. The Cancer Genome Atlas (TCGA) project, for example, has generated large amounts of data by applying the different “omics” technologies to study organ-site specific cancer specimens [2]–[5]. The TCGA data include somatic mutations, gene expression, methylation and copy number variation, which together with clinical information from the patients represent an important resource for the development of new strategies for diagnostic and therapeutic interventions as well as providing baseline data for more detailed studies of specific genes and pathways [2]–[5].

These genome-wide data have been used to identify genes that are altered in cancer. These alterations typically occur in tumor suppressor genes like p53 or oncogenes like KRAS. Alterations in tumor suppressor genes usually lead to the loss of function of the respective proteins while alterations in oncogenes lead to increased or altered activity either due to higher expression or activating mutations. Although there are genes that are frequently altered in cancer, a striking example being p53, one of the main conclusions from the first large-scale studies is that the tumorigenic process is driven by alterations in a variety of genes, both individually and in combination, depending on the individual context of the patient, among other factors [2]–[7].

One important issue in the analysis of these “omics” data sets is how to measure the impact of all genetic alterations found in a cohort of samples. What is required for such an impact study is a gene-specific score that is both qualitative (indicating if a gene is a suppressor, an oncogene, either or both) and quantitative (indicating the frequency of alterations for that gene in a given set of tumors). Previous attempts to generate scores for cancer genes have used a single type of data, either mutation frequency or expression pattern [6], [8]. More recently, Volgestein et al. [1] proposed a strategy that takes into account both the type of somatic mutations (recurrent missense for oncogenes and inactivating mutations for tumor suppressors) and their frequency (they adopted a 20% rule, i.e., those types of mutations had to appear in at least 20% of the analyzed samples). Although this strategy may efficiently identify the most common driver mutations in tumors, it does not explore the whole spectrum of genetic/epigenetic alterations that generate the characteristic genetic heterogeneity in tumors. Another approach has involved the calculation of the number of non-redundant samples in which a given gene or group of genes is altered. Although this strategy has been widely used, as for example in the CBio Cancer Genome Portal [9], it does not discriminate between oncogenic and tumor suppressing alterations and does not allow the user to provide different weights for the type of genetic alteration found.

Here we propose the S-score, which integrates information on mutation status, expression pattern, methylation status and copy number to produce a unique value directly proportional to the frequency in which a given gene is altered in a cancer type. The critical value of this method is that it facilitates the identification of predicted cancer genes, rank orders them to prioritize them for future in-depth analysis and indicates which features (e.g., mutation, expression, methylation, copy number change and combinations thereof) should be further investigated. As a proof of principle, here the S-score method was applied to data derived from the Cancer Genome Atlas (TCGA) project for GBM, colorectal, ovary and breast tumors.

## Material and Methods

### Data source

Expression z-scores, methylation and GISTIC CNV (copy number variation) data were obtained from the cBIO portal by using the CGDS-R package, which provides a basic set of functions for querying the Cancer Genomic Data Server (CGDS) via the R platform for statistical computing (http://cran.r-project.org/web/packages/cgdsr/index.html). Somatic mutation data was obtained from the COSMIC database [10] and from a local compilation of all somatic mutations found in the literature. Thresholds for all types of data are discussed below. Clinical data for all samples were obtained from the TCGA web site (https://tcga-data.nci.nih.gov/tcga/tcgaHome2.jsp).

### CNV amplification and deletion

Putative copy-number calls on samples were determined using GISTIC [9]. The published GISTIC thresholds used in the present study were: homozygous deletion, < =  −2; deletion, > −2 to < =  −1; neutral > −1 to < +1; gain, > =  +1 to <2; and amplification, > =  2. Boxplots were generated using ggplot2, a graphics tool for the R statistical package.

### Expression analysis

**Figure 1:**
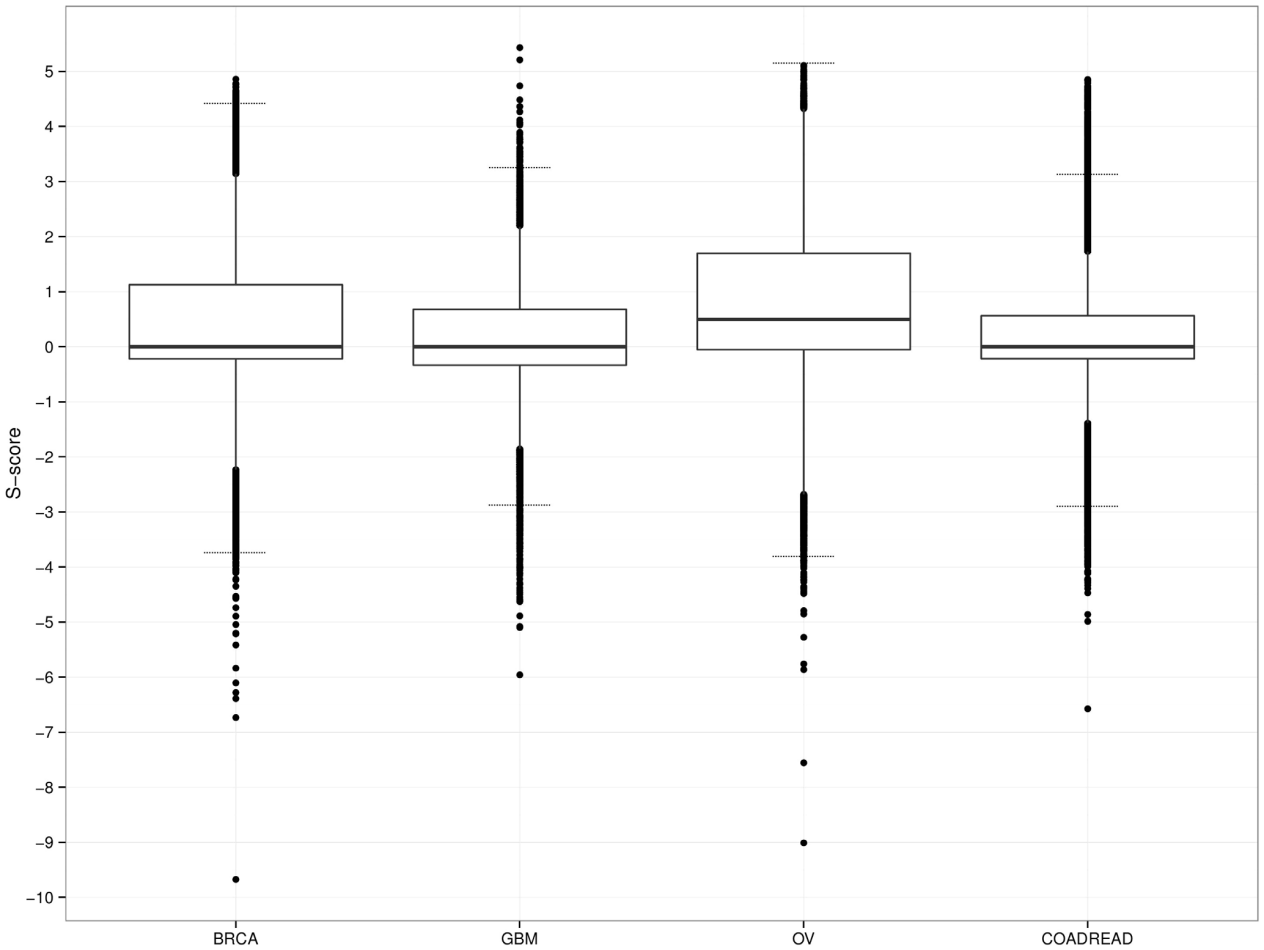
S-score distribution for the four types of tumors analyzed here. Transversal gray lines indicate a Z-score threshold equal to 3. GBM, glioblastoma; OV, ovarian cancer; BRCA, breast cancer; and COADREAD, colorectal cancer.

**Figure 2:**
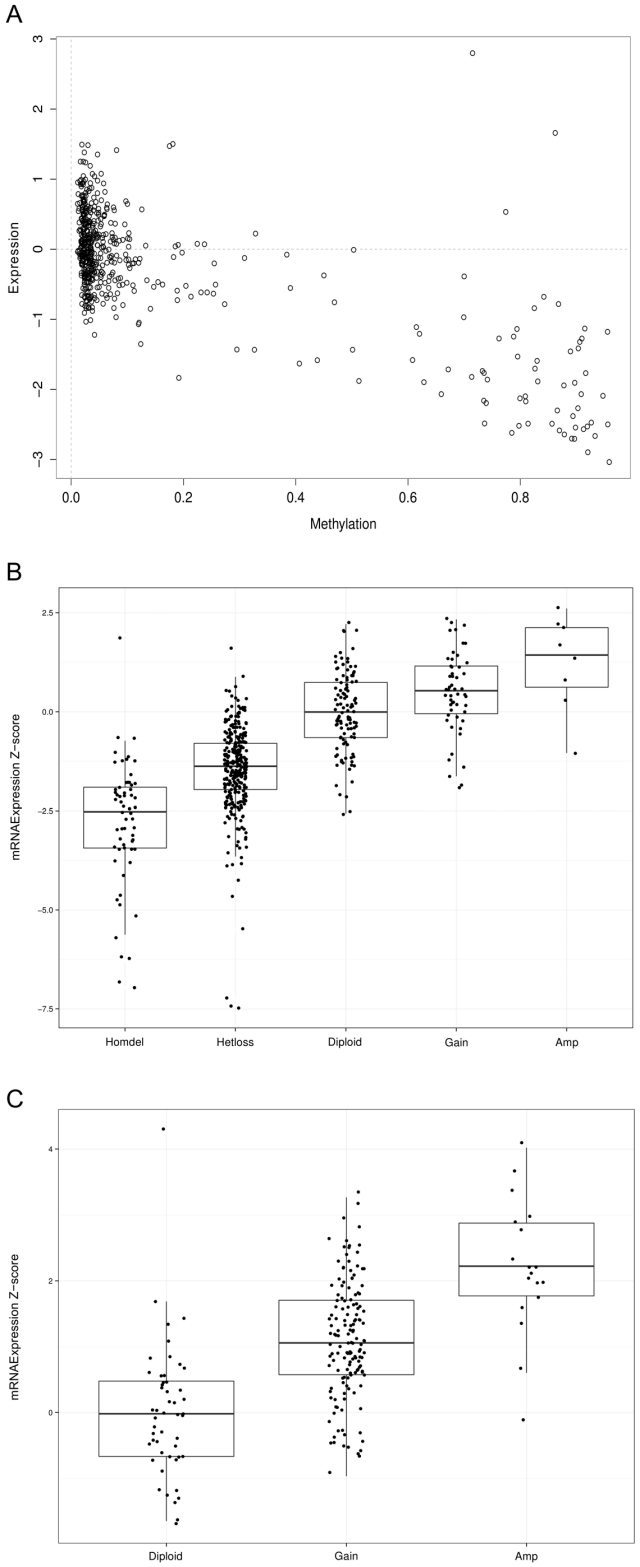
Examples of genes of interest. Each data point corresponds to a sample. (A) Scatter plot showing expression (Y axis) and methylation status (X axis) for TMEM101 in the set of ovarian tumors from TCGA. (B) Scatter plot showing expression (Y axis) and copy number status for FBXO25 for ovarian cancer from TCGA. Based on Gistic values, samples were split in different categories (X axis). See Methods for Gistic thresholds. (C) Scatter plot showing expression (Y axis) and copy number status for ACTR5 in colon tumors from TCGA. Based on Gistic values, samples were split in different categories (X axis).

Expression data from the cBio portal was used in the analysis presented here [9]. The expression level given is the relative expression of a given gene compared to the expression of that gene in a reference population (either adjacent normal samples or tumors that are diploid for that gene). Up and down-regulation were inferred by the Z-score of that expression level, i.e., the number of standard deviations from the mean of expression in the reference population. The same expression data was used in the calculation of the S-score in Figure 1 and also as an independent dataset in Figure 2.

### Somatic mutations

**Figure 3:**
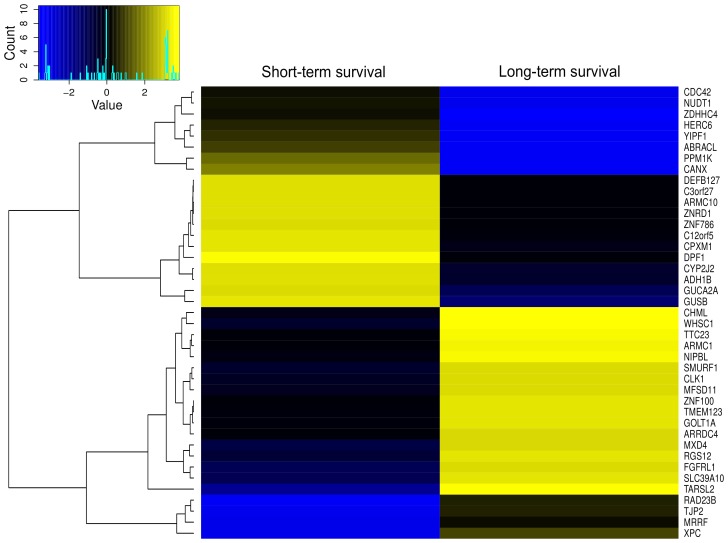
Genes associated with short and long-term survival. A heatmap plot showing genes with S-scores significantly different between short-term and long-term survival patients with ovarian tumors. Blue is indicative of negative S-score while yellow is indicative of positive S-score.

**Figure 4:**
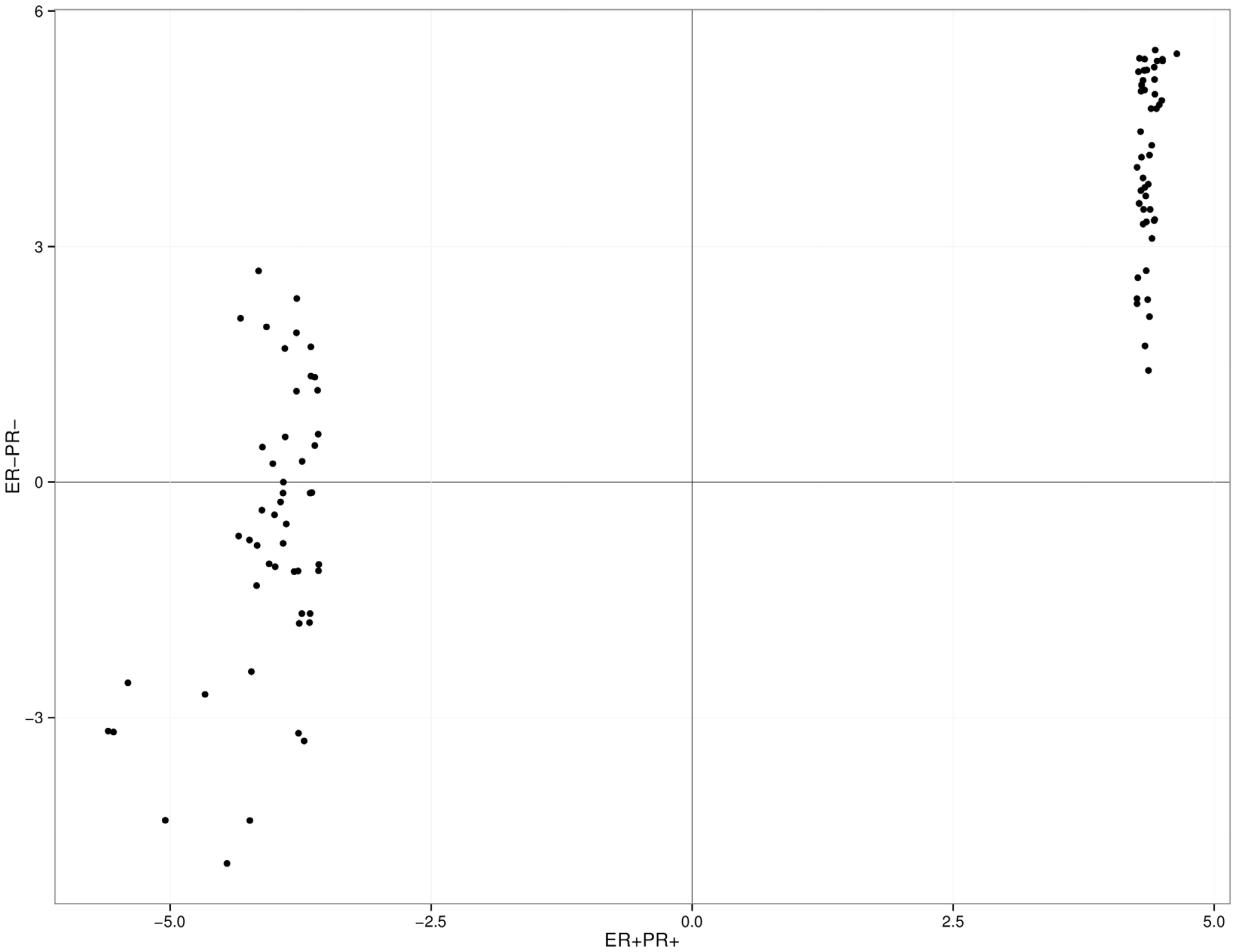
Oncogenes and suppressors in two breast tumor sub-types. S-score comparison for the 50 top oncogenes and 50 top tumor suppressors between ER-PR- and ER+PR+ breast cancer subtypes. Each datapoint is a gene. X and Y axes represents the S-scores for ER+PR+ and ER-PR- sub-types, respectively.

**Figure 5:**
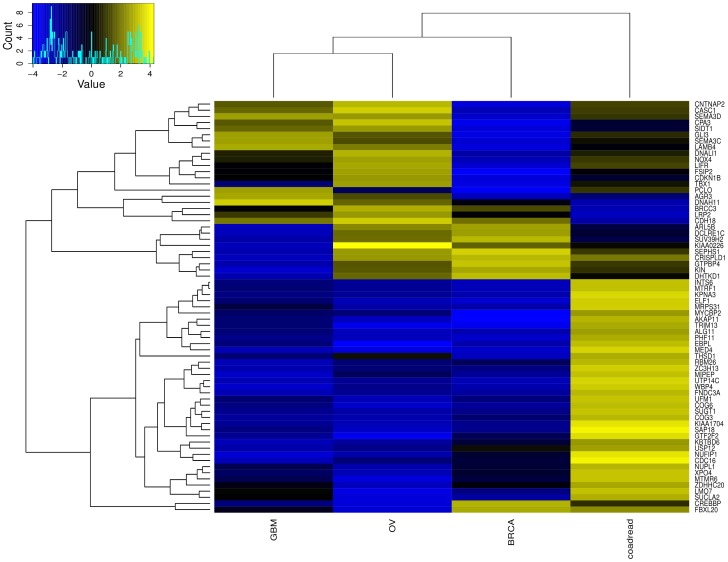
Genes classified as oncogenes and suppressors in different tumor types. Genome-wide analysis of genes behaving as tumor suppressor in one tumor type and oncogene in a different tumor type. Sixty-seven genes with S-score<−2.5 in one tumor type and S-score >2.5 in a different tumor type were selected and a heatmap showing their S-score for all tumor types is presented. Blue represents negative S-scores while yellow represents positive S-score.

To calculate the S-score, we only considered nonsense mutations (variable ns in the equations presented in the text) found for the respective gene in that tumor type. The 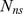 variable was stratified to two possible situations: 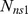 where only nonsense mutations occurring in tumor samples from TCGA were considered and 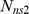 where nonsense mutations occurring in the same tumor type (all samples available in COSMIC) were considered. 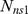 was used for data presented in Figures 3 and 4 while 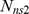 was used for the analysis presented in Figure 1, Figure 5 and Table 1.

**Table 1:** Known cancer genes have extreme S-scores.

	GBM	OV	BR	CR
**Real Set**	19	54	56	22
**10,000 Simulated Sets**	8.08 (2.49–13.66)	34.07(23.76–44.38)	23.07(14.27–31.87)	9.15(3.19–15.11)
				
**p-value**	0.0002	0.0001	<0.0001	0.0001

Number of genes (Real Set) with S-score >2 or <−2 in the 138 cancer gene list from Volgestein et al. [1]. Numbers in the “10,000 Simulated Sets” row correspond to average number of genes with S-score >2 or <−2 in 10,000 sets containing 138 genes randomly selected. Between parentheses is the interval corresponding to the average +/− 2× standard deviation. P-value of the difference between real and simulated sets is shown in the last row.

## Results and Discussion

The S-score is given by the equation #1:
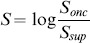
(1)where,
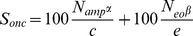
(2)and
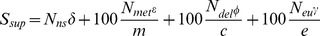
(3)


where,


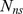
  =  number of nonsense mutations for the respective gene.


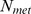
 =  number of samples in which the respective gene is methylated.


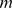
 =  total number of samples informative for methylation analysis.


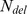
 =  number of samples in which the respective gene is deleted


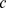
 =  total number of samples informative for CNV analysis.


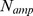
 =  number of samples in which the respective gene is amplified.


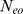
 =  number of samples in which the respective gene is over-expressed.


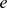
 =  total number of samples informative for gene expression analysis.


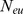
 =  number of samples in which the respective gene is under-expressed.


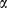
 =  index for amplification.


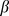
 =  index for over-expression.


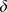
 =  index for nonsense mutations.


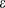
 =  index for methylation.


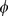
 =  index for deletions.


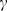
 =  index for under-expression.

In case 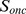
<1 and 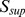
>1, then 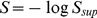
(4)


In case 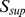
<1 and 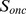
>1, then
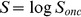
(5)


In case 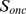 and 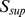 are both smaller than 1, then 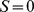. Throughout this report, log is a representation of log_2_.

The use of log in equation #1 allows the S-score to range from negative (indicative of tumor suppressing or reduced gene activity) to positive (indicative of oncogene or increased gene activity) values. The S-score as a ratio between 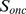 (equation #2) and 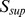 (equation #3) also aims to give more value to those genes that present an exclusive pattern of either tumor suppressing or oncogene activity in a respective tumor type. Another important issue to emphasize is that each type of data, CNV, mutation, expression and methylation, is treated independently and has a proportional weight given by the numerical index associated to each data type.

The S-score method was tested using data from the TCGA project for four types of tumors: glioblastoma (GBM), colorectal tumor, breast tumor and ovary tumor. A critical parameter in calculating the S-score is the numerical index used for each type of data. To find the best index values for the parameters in equations #2 and #3, two values for each index were tested. In all scenarios, more weight was given to nonsense mutations due to the fact that this type of alteration usually leads to a significant decrease in the function of the respective protein. Furthermore, in all scenarios methylation was not used due to quality control issues.

A list of 138 cancer genes identified by Volgestein et al [1] was used as a benchmark to evaluate which set of indexes would select more known oncogenes and tumor suppressors. Although this list was compiled using data from several tumor types and here we have only analyzed four tumor types, we believe our analysis is comprehensive enough for such test. For each tumor type analyzed here the number of genes with S-score <−2 or >+2 was calculated for each scenario (Table S1). To test for a possible enrichment, a Monte Carlo simulation was performed where random sets of 138 genes (out of all known human genes with an S-score for the respective tumor) were selected and the number of extreme S-scores calculated. Among all tested scenarios, the one with a higher value for nonsense mutations (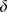 = 5) and a value of 0.5 for all other indexes promoted the most significant enrichment of known cancer genes for all tumor types (Table S1). Furthermore, to avoid any bias due to an arbitrary threshold (S-score <−2 or >+2), we used a new threshold for each tumor type defined as the S-score with a Z score of 2 (average of all S-scores plus or minus two standard deviations) (Table S2). The same set of indexes, as with the previous analysis, showed the higher enrichment of known cancer genes. This set of indexes (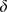 = 5; 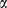 = 0.5; 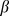 = 0.5; 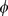 = 0.5 and 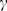 = 0.5) was then used for all other studies.

To gain more information on the predictive capacity of the S-score method, a different benchmark strategy was performed to define “positive predictive value” and “negative predictive value” for each tumor type. A thousand random sets of 50 genes were selected from the list of 138 genes from Volgestein et al. [1] and were used to calculate the average number of true positives and false negatives. In a similar fashion, one thousand random sets of 50 genes were selected from all human genes (minus the 138 cancer genes) were selected and used to calculate the average number of true negatives and false positives for each tumor type. These values are shown in Table S3 It is worth mentioning, however, that the list of cancer genes from Volgestein et al. [1] is not the golden standard for this type of analysis since it contains several genes that are either oncogenes or suppressors in tumor types different than the ones analyzed here. These features likely underestimate the predictive capacity of the S-score method.

These previous analyses show that the S-score method is able to identify *bona fide* oncogenes and tumor suppressors. Data shown in Table 1 confirms that the compilation of cancer genes from Volgestein et al. [1] is biased towards extreme S-scores (>+2 or <−2). When a normalized threshold is used (S-scores representing the average S-score plus or minus two standard deviations) the same pattern is observed (Table S4).

Figure 1 plots the distribution of the S-scores for all human genes in each tumor type. Those human genes with S-scores that were positive or negative extremes (Z score >3) in at least one tumor type are listed in Table S5. As a confirmation of this method, previously known tumor suppressors and oncogenes show extreme S-score values for these types of tumors. In GBM, for example, the gene with the highest S-score is EGFR. Other genes with high positive S-scores include those that are mapped to the same locus as EGFR (like SEC61G, LANCL2 and ECOP) and are therefore amplified together with EGFR. While these genes are not necessarily causally involved in the tumorigenic process, they represent bona fide genetic alterations in the tumor type that might provide new therapeutic and diagnostic opportunities, as reported for passenger genes deleted in tumors [11], and as such should be reported. The efficiency of our method is also illustrated at the other end of the S-score distribution. Among the genes with the most negative S-scores are well known tumor suppressor genes like CDKN2A (the most negative S score for GBM), PTEN, NF1 and RB1. The S-scores for all human genes in the four tumor types is provided in Table S6.

One utility of the S-Score system is that it allows easy identification of genes of interest for additional analysis. For example, consider the genes FBXO25 (S-Score = −3.18 in ovarian cancer), TMEM101 (S-Score = −1.6 in ovarian cancer) and ACTR5 (S-Score  =  +3.69 in colon cancer) that are classified by our analysis as suppressor, putative suppressor and oncogene, respectively. Evaluation of plots of expression vs. copy number or methylation for these genes, as appropriate (Figure 2) readily identifies these genes as having an identifiable fraction of TCGA cases associated with reduced copy number and reduced expression (candidate suppressor gene), reduced expression and increased methylation (candidate silenced suppressor gene) and increased copy number and increased expression (candidate oncogene), respectively. To illustrate the usefulness of such strategy plots for known oncogenes and suppressors are provided as Figures S1-S3. This type of more detailed classification will then facilitate follow-up studies by providing a prioritization of the genes, based on score, for further analysis. None of the three genes above have been previously identified as been involved in the development of the respective tumor types.

The S-score also allows for a direct comparison between samples classified differently according to a biological and/or clinical parameter. To illustrate this application, the samples in the TCGA high-grade serous ovarian cancer data were divided into quartiles according to overall survival. We then calculated the S-score for all human genes using the samples belonging to the first (shortest survival) and last (longest survival) quartile of the survival distribution. A comparison of S-scores calculated from the two groups allowed us to identify putative oncogenes (with positive S-scores) and putative tumor suppressor genes (with negative S-scores) associated with either the shortest or the longest survival (Figure 3). Several of the genes identified are known markers for survival. For example, CDC42 inhibition has been associated with longer survival in mice with prostate cancer xenografts [12]. Another example is CANX whose down-regulation has been associated with longer survival in GBM patients [13]. Furthermore, genetic variants of RGS12 have been associated with survival in late-stage non-small cell lung cancer [14]. Another interesting gene is TJP2 whose over-expression has been associated with long-term survival in GBM [15], in agreement with the pattern shown in Figure 3.

Among the genes identified by this scoring system to be associated with survival, the most interesting are those with opposite classifications (positive and negative scores) in the shortest or the longest survival quartiles. We found that glucoronidase B (GUSB) had a positive score (+3.04, indicative of oncogene) for the shortest survival group and a negative score (−1.40, indicative of tumor suppressor) for the longest survival group. Glucuronidases are known for being involved in the spreading of tumor cells from the primary site [16] and GUSB has been recently included in a signature for predicting lymph node metastasis in cervical cancer [17]. The S-score method confirms the idea that GUSB has an oncogenic function in the more aggressive tumors (shortest survival). However, its negative S-score in the less aggressive tumors indicates that the loss of GUSB might also drive ovarian cancer development with the resulting tumors being less aggressive. An interesting finding in our analysis is the association of RAD23B and XPC, both with negative S-scores, with short-term survival (Figure 3). Proteins encoded by these genes form a complex involved in DNA-damaged repair. A number of other genes with opposite S-scores in the shortest and the longest survival groups are presented in Figure 3. These genes may represent potential prognostic biomarkers as well targets for the development of new therapies.

To further explore the potential of the S-score system to identify genes related to different clinical parameters, breast cancer patients from the TCGA cohort were divided according to two hormonal subtypes: ER+PR+ and ER-PR- (ER: Estrogen receptor; PR: progesterone receptor). Data from patients in each subtype were then used to calculate the S-scores for all human genes. While the oncogenes in the two subtypes are basically the same, a much larger discordance is observed for tumor suppressor genes. This is shown in the scatter plot in Figure 4, which contains the top 50 putative oncogenes and 50 putative suppressors (classified according to the ER+PR+ subtype). While all the oncogenes in the ER+PR+ subtype (S-score around 4) are also classified as oncogenes in the ER-PR- subtype (S-score ranging from 1.42 to 5.50), the tumor suppressors in the ER+PR+ (S-score around -4) have a different classification in the ER-PR- subtype (S-score ranging from -4.85 to 2.69). In fact, a large fraction of the suppressors in the ER+PR+ subtype were classified as oncogenes in the other subtype (Figure 4). These results suggest that the differences in biological and clinical features between these two breast cancer subtypes may be due to differences in their tumor suppressors genes. These gene signatures represent an opportunity for the development of targets for new diagnostic, prognostic and therapeutic approaches.

The S-score method was also used in a genome-wide search for genes that can behave as suppressor in one tumor type and oncogenes in a different tumor type. In the last few years some genes have been shown to present such pattern. NOTCH1, for example, is a known oncogene for T cell acute lymphoblastic leukemia [18]–[19] but also presents tumor suppressive activity in skin tumors [20] and hepatocarcinoma [21]. Using a set of stringent criteria (S-score>2.5 in one tumor type and S-score<−2.5 in a different tumor type), we found 65 genes that showed oncogenic and tumor suppressive activities in different tumor types (among the four types analyzed here). Our analysis identified LMO7 as a gene behaving as tumor suppressor and oncogene. This gene has been reported to be down-regulated in lung cancer [22] and mice lacking this gene have an increased susceptibility to spontaneous lung cancer [23]. On the other hand, the gene seems to be an oncogene in both breast [24] and liver cancer [25]. Another interesting candidate is USP12, a gene coding for a deubiquitinase. Recently, USP12 has been shown to be a positive regulator of androgen receptor acting in a pro-proliferative manner in prostate cancer [26]. USP12 can also act as a tumor suppressor by negatively regulating AKT activation and thus promoting apoptosis [27]. Further analyses are needed to fully explore all genes shown in Figure 5. It is important to emphasize that NOTCH1 has not appeared in our list due to the fact that we haven't used leukemia data in our studies.

A drawback of the S-score method, which is a limitation in any attempt to establish this type of scoring system, is the lack of an index for activating mutations occurring in oncogenes. For example, activating mutations in KRAS are known to be a determinant factor for many tumor types [28]. Although the S-score for KRAS was positive for three out of four tumors analyzed here, our method was not able to fully measure the impact of these types of activating mutations in oncogenes. One possibility would be the use of missense mutations, as argued by Volgestein et al. [1]. One problem with missense mutations, however, is how to evaluate their impact at protein level, whether they are activating, inactivating or neutral. Although there are computational tools aimed to infer the effect of a missense mutation at the protein level, we still think that their performance in general is poor [29]. However, as we improve our understanding of the nature of missense mutations, these types of genetic alterations can be incorporated in the calculation of the S score.

To make the S-score system more useful to the community, a web portal is provided at http://www.bioinformatics-brazil.org/S-score with genome-wide scores available for download as well as a retrieval system for customized queries. Furthermore, users can modify the values for all the parameters in equations #2 and #3 and generate S-scores for all known human genes. A list of all TCGA samples from each tumor type used in this study is provided as Table S7.

## Supporting Information

Figure S1 **Expression X methylation plot for the known tumor suppressor MGMT.** Each data point represents a GBM sample. Data shows the silencing of MGMT in several GBM samples.
(TIF)

Figure S2 **Expression X copy number variation plot for the known tumor suppressor CDKN2A.** Each data point represents a GBM sample. Categories of copy number variation were defined by the GISTIC classification. Homdel  =  homozygous deletion; Hetloss  =  loss of heterozygosis.
(TIF)

Figure S3 **Expression X copy number variation plot for the known oncogene ERBB2.** Each data point represents a breast tumor sample. Categories of copy number variation were defined by the GISTIC classification. Hetloss  =  loss of heterozygosis; Amp  =  amplification.
(TIF)

Table S1 **Selection of indexes for parameters in the S-score equations.** Each row represents a scenario of values for indexes. The number in parenthesis corresponds to the number of genes above the threshold (S-score >+2 or S-score <−2) in the real set of 138 genes from Volgestein et al. [1]. Numbers in each cell correspond to the number of simulated sets in which the number of genes with S-scores above the threshold is equal or higher the corresponding number in the real set (number in parenthesis).
(DOCX)

Table S2 **Selection of indexes for parameters in the S-score equations.** Each row represents a scenario of values for indexes. Number in parenthesis corresponds to the number of genes above the threshold (S-score values corresponding to the average plus or minus two standard deviations) in the real set of 138 genes from Volgestein et al. [1]. Numbers in each cell correspond to the number of simulated sets in which the number of genes with S-scores above the threshold is equal or higher the corresponding number in the real set (number in parenthesis).
(DOCX)

Table S3 A thousand random sets of 50 genes were selected from the list of 138 genes from Volgestein et al. [1] and were used to calculate the average number of true positives and false negatives. Positive Predictive Value (PPV) was calculated by the following equation: true positive/true positive + false positive. In a similar fashion, one thousand random sets of 50 genes were selected from all human genes (minus the 138 cancer genes) and used to calculate the average number of true negatives and false positives for each tumor type. Negative predictive value was calculated by the following equation: true negative/true negative + false negative.
(DOCX)

Table S4 **Known cancer genes have extreme S-scores.** Number of genes (Real Set) with S-scores greater than the average plus two standard deviations (Z score = 2) or smaller than the average minus two standard deviations (Z score  =  −2) in the 138 cancer gene list from Volgestein et al. [1]. Numbers in the “10,000 Simulated Sets” row correspond to average number of genes with S-score above or below the threshold in 10,000 sets containing 138 genes randomly selected. Between parentheses is the interval corresponding to the average +/− 2× standard deviation. P-value of the difference between real and simulated sets is shown in the last row.
(DOCX)

Table S5 **Correlation between Z-score and S-score for BRCA tumor.** Each spreadsheet lists all human genes with S-scores that were positive or negative extremes (Z-score >3).
(XLSX)

Table S6 **S-scores for all human genes.** For each of the four tumor types analyzed here, all human genes are alphabetically listed with their corresponding S-scores.
(XLSX)

Table S7 **Identification of all TCGA samples used in this study.** Identification number for all TCGA samples used in this study.
(XLS)
