# Supplementary figures and images for: S-Score: A Scoring System for the Identification and Prioritization of Predicted Cancer Genes
Source: PLoS One. 2014 Apr 7;9(4):e94147. doi: 10.1371/journal.pone.0094147 (PMC3978018; doi:10.1371/journal.pone.0094147)

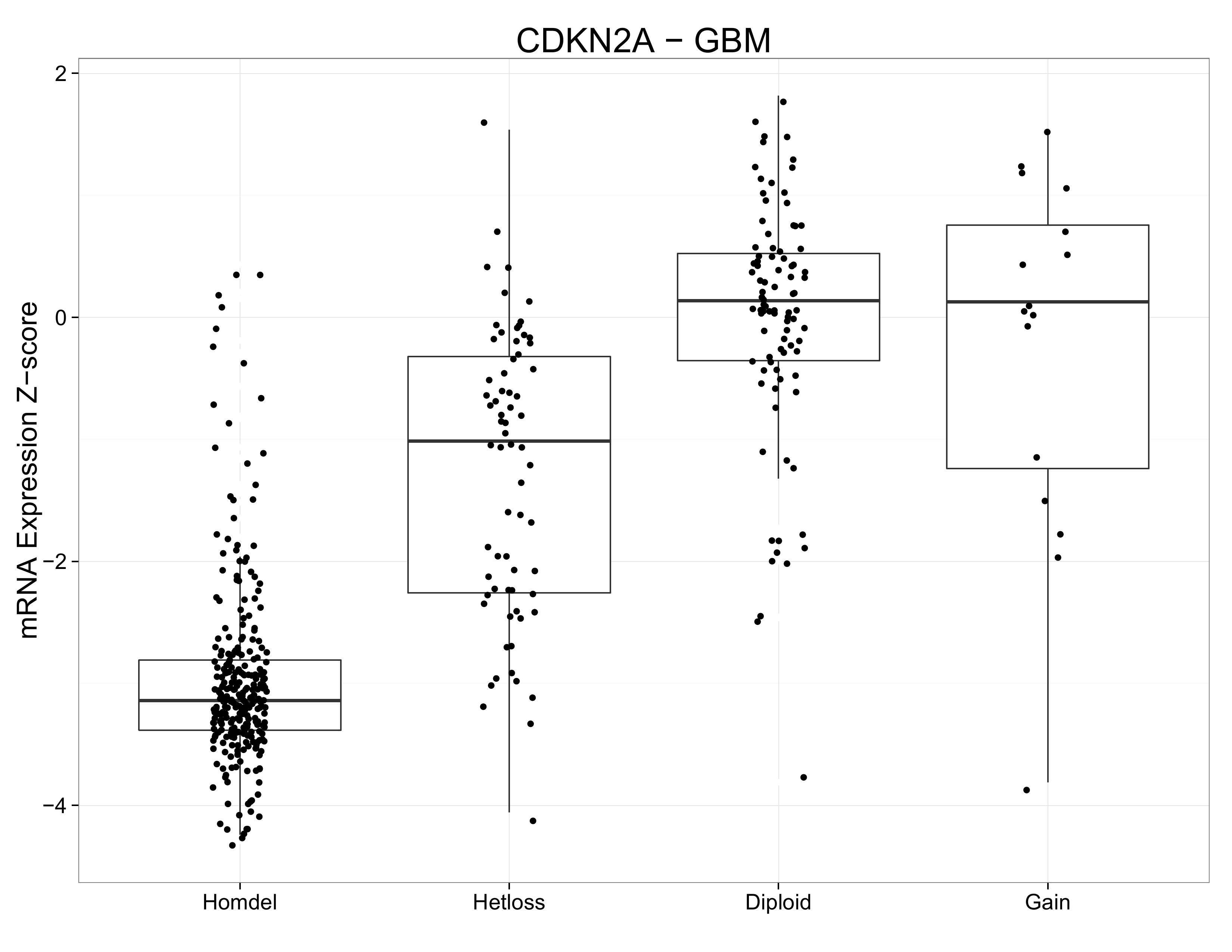

Supplement: Figure S2 — Expression X copy number variation plot for the known tumor suppressor CDKN2A. Each data point represents a GBM sample. Categories of copy number variation were defined by the GISTIC classification. Homdel = homozygous deletion; Hetloss = loss of heterozygosis. (TIF) [file pone.0094147.s002.tif]

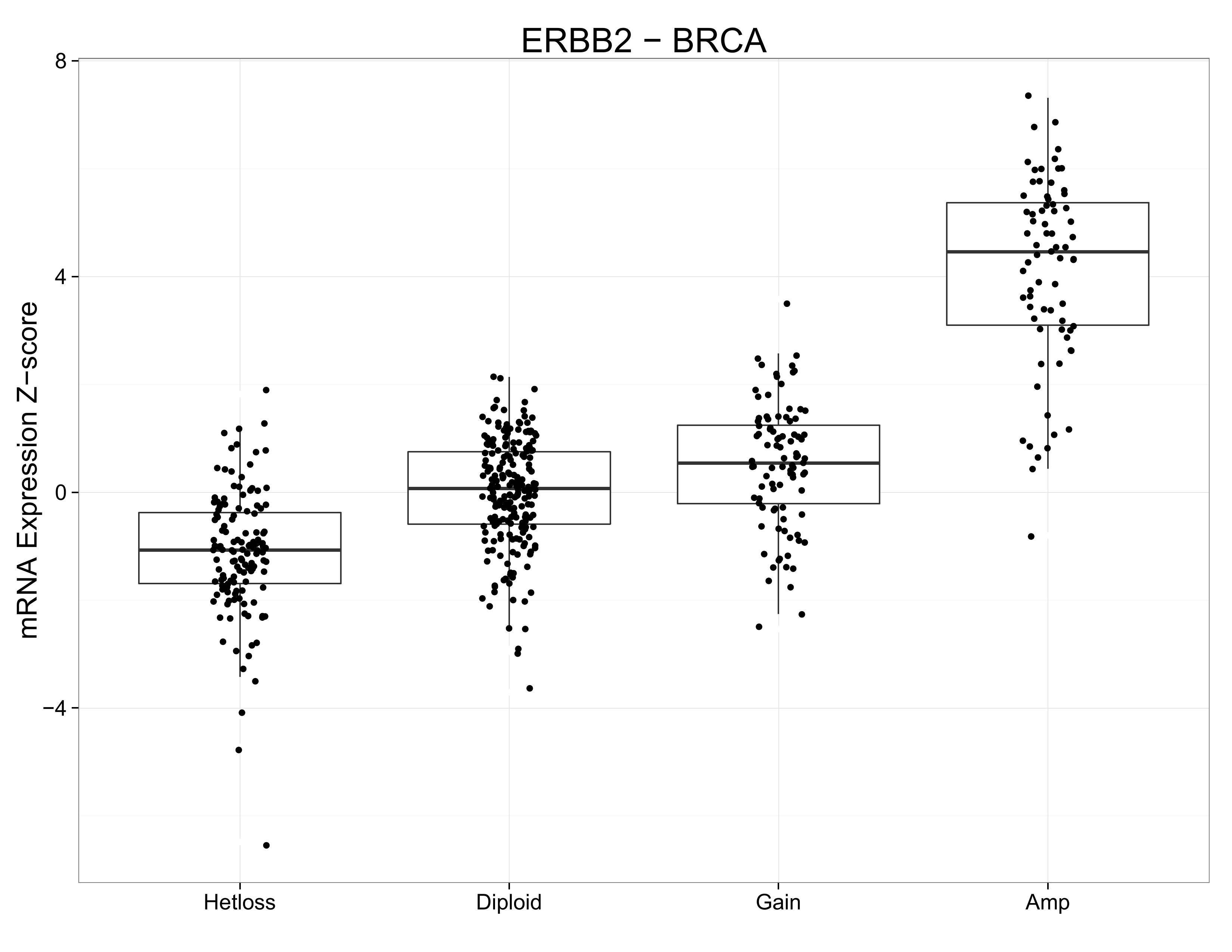

Supplement: Figure S3 — Expression X copy number variation plot for the known oncogene ERBB2. Each data point represents a breast tumor sample. Categories of copy number variation were defined by the GISTIC classification. Hetloss = loss of heterozygosis; Amp = amplification. (TIF) [file pone.0094147.s003.tif]
