# Supplementary material for: S-Score: A Scoring System for the Identification and Prioritization of Predicted Cancer Genes
Source: PLoS One. 2014 Apr 7;9(4):e94147. doi: 10.1371/journal.pone.0094147 (PMC3978018; doi:10.1371/journal.pone.0094147)
Supplement: Table S1 — Selection of indexes for parameters in the S-score equations. Each row represents a scenario of values for indexes. The number in parenthesis corresponds to the number of genes above the threshold (S-score >+2 or S-score <−2) in the real set of 138 genes from Volgestein et al. [1]. Numbers in each cell correspond to the number of simulated sets in which the number of genes with S-scores above the threshold is equal or higher the corresponding number in the real set (number in parenthesis). (DOCX) [file pone.0094147.s004.docx]

|  | Breast | GBM | Ovary | Colorectal |
| --- | --- | --- | --- | --- |
| δ=5; all other indexes = 0.5 | 0 (56) | 2 (19) | 1 (54) | 1 (22) |
| δ=5; α=1; φ=1; all other indexes =0.5 | 0 (55) | 5 (23) | 488 (60) | 63 (22) |
| δ=5; β=1; γ=1; all other indexes =0.5 | 0 (58) | 2 (38) | 111 (54) | 9 (34) |
| δ=3; all other indexes = 0.5 | 0 (49) | 18 (18) | 23 (50) | 1 (22) |

**Supplementary Table S1: Selection of indexes for parameters in the S-score equations**. Each row represents a scenario of values for indexes. The number in parenthesis corresponds to the number of genes above the threshold (S-score >+2 or S-score <-2) in the real set of 138 genes from Volgestein et al. [1]. Numbers in each cell correspond to the number of simulated sets in which the number of genes with S-scores above the threshold is equal or higher the corresponding number in the real set (number in parenthesis).
