# Supplementary material for: S-Score: A Scoring System for the Identification and Prioritization of Predicted Cancer Genes
Source: PLoS One. 2014 Apr 7;9(4):e94147. doi: 10.1371/journal.pone.0094147 (PMC3978018; doi:10.1371/journal.pone.0094147)
Supplement: Table S2 — Selection of indexes for parameters in the S-score equations. Each row represents a scenario of values for indexes. Number in parenthesis corresponds to the number of genes above the threshold (S-score values corresponding to the average plus or minus two standard deviations) in the real set of 138 genes from Volgestein et al. [1]. Numbers in each cell correspond to the number of simulated sets in which the number of genes with S-scores above the threshold is equal or higher the corresponding number in the real set (number in parenthesis). (DOCX) [file pone.0094147.s005.docx]

|  | Breast | GBM | Ovary | Colorectal |
| --- | --- | --- | --- | --- |
| δ=5; all other indexes = 0.5 | 0 (33) | 2 (19) | 0 (24) | 2 (20) |
| δ=5; α=1; φ=1; all other indexes =0.5 | 0 (28) | 20 (17) | 1 (18) | 51 (18) |
| δ=5; β=1; γ=1; all other indexes =0.5 | 0 (30) | 163 (12) | 0 (23) | 115 (15) |
| δ=3; all other indexes = 0.5 | 0 (29) | 6 (18) | 0 (21) | 5 (20) |

**Supplementary Table S2: Selection of indexes for parameters in the S-score equations**. Each row represents a scenario of values for indexes. Number in parenthesis corresponds to the number of genes above the threshold (S-score values corresponding to the average plus or minus two standard deviations) in the real set of 138 genes from Volgestein et al. [1]. Numbers in each cell correspond to the number of simulated sets in which the number of genes with S-scores above the threshold is equal or higher the corresponding number in the real set (number in parenthesis).
