# Supplementary material for: S-Score: A Scoring System for the Identification and Prioritization of Predicted Cancer Genes
Source: PLoS One. 2014 Apr 7;9(4):e94147. doi: 10.1371/journal.pone.0094147 (PMC3978018; doi:10.1371/journal.pone.0094147)
Supplement: Table S3 — A thousand random sets of 50 genes were selected from the list of 138 genes from Volgestein et al. [1] and were used to calculate the average number of true positives and false negatives. Positive Predictive Value (PPV) was calculated by the following equation: true positive/true positive + false positive. In a similar fashion, one thousand random sets of 50 genes were selected from all human genes (minus the 138 cancer genes) and used to calculate the average number of true negatives and false positives for each tumor type. Negative predictive value was calculated by the following equation: true negative/true negative + false negative. (DOCX) [file pone.0094147.s006.docx]

|  | Positive Predictive  Value | Negative Predictive Value |
| --- | --- | --- |
| BREAST | 0.71 | 0.58 |
| COLORECTAL | 0.71 | 0.52 |
| GBM | 0.70 | 0.52 |
| OVARY | 0.61 | 0.61 |

**Supplementary Table S3:** A thousand random sets of 50 genes were selected from the list of 138 genes from Volgestein et al. [1] and were used to calculate the average number of true positives and false negatives. Positive Predictive Value (PPV) was calculated by the following equation: true positive/true positive + false positive. In a similar fashion, one thousand random sets of 50 genes were selected from all human genes (minus the 138 cancer genes) and used to calculate the average number of true negatives and false positives for each tumor type. Negative predictive value was calculated by the following equation: true negative/true negative + false negative.
