# Supplementary material for: S-Score: A Scoring System for the Identification and Prioritization of Predicted Cancer Genes
Source: PLoS One. 2014 Apr 7;9(4):e94147. doi: 10.1371/journal.pone.0094147 (PMC3978018; doi:10.1371/journal.pone.0094147)
Supplement: Table S4 — Known cancer genes have extreme S-scores. Number of genes (Real Set) with S-scores greater than the average plus two standard deviations (Z score = 2) or smaller than the average minus two standard deviations (Z score = −2) in the 138 cancer gene list from Volgestein et al. [1]. Numbers in the “10,000 Simulated Sets” row correspond to average number of genes with S-score above or below the threshold in 10,000 sets containing 138 genes randomly selected. Between parentheses is the interval corresponding to the average +/− 2× standard deviation. P-value of the difference between real and simulated sets is shown in the last row. (DOCX) [file pone.0094147.s007.docx]

|  | **GBM** | **OV** | **BR** | **CR** |
| --- | --- | --- | --- | --- |
| **Real Set** | 19 | 24 | 33 | 20 |
| **10,000**  **Simulated Sets** | 7.56(2.25-12.87) | 7.41 (2.12-12.70) | 8.39 (2.77-14.01) | 8.36(2.70-14.01) |
|  |  |  |  |  |
| **p-value** | 0.0002 | <0.0001 | <0.0001 | 0.0002 |

**Supplementary Table S4**: **Known cancer genes have extreme S-scores.** Number of genes [Real Set] with S-scores greater than the average plus two standard deviations [Z score=2] or smaller than the average minus two standard deviations [Z score = -2] in the 138 cancer gene list from Volgestein et al. [1]. Numbers in the “10,000 Simulated Sets” row correspond to average number of genes with S-score above or below the threshold in 10,000 sets containing 138 genes randomly selected. Between parentheses is the interval corresponding to the average +/- 2x standard deviation. P-value of the difference between real and simulated sets is shown in the last row.
